# Characterization of *SSBP1*-related optic atrophy and foveopathy

**DOI:** 10.1038/s41598-021-98150-1

**Published:** 2021-09-21

**Authors:** Isabelle Meunier, Béatrice Bocquet, Sabine Defoort-Dhellemmes, Vasily Smirnov, Carl Arndt, Marie Christine Picot, Hélène Dollfus, Majida Charif, Isabelle Audo, Hélèna Huguet, Xavier Zanlonghi, Guy Lenaers

**Affiliations:** 1 National reference centre for inherited sensory diseases, University Hospital of Montpellier, University of Montpellier, Montpellier, France; 2 Sensgene Care Network, Strasbourg, France; 3 Institute for Neurosciences of Montpellier, Inserm, University of Montpellier, Montpellier, France; 4 Department of Neuro-Ophthalmology and Electrophysiology, Robert Salengro Hospital, Lille, France; 5 Department of Ophthalmology, University Hospital of Reims, Reims, France; 6 Clinical Investigation Center (CIC) and Clinical Research and Epidemiology Unit (URCE), Montpellier, France; 7 Department of Ophthalmology, National Center for Rare Disorders in Ophthalmic Genetics CARGO, Strasbourg Hospital, Strasbourg, France; 8 Genetics and Immuno-Cell Therapy Team, Mohammed First University, Oujda, Morocco; 9 CNRS, INSERM, Institut de la Vision, Sorbonne Université, Paris, France; 10 DHU Sight Restore, INSERM-DHOS CIC1423, CHNO des Quinze-Vingts, Paris, France; 11 Clinic Jules Verne, Nantes, France; 12 Department of Ophthalmology, University Hospital of Rennes, Rennes, France; 13 UMR CNRS 6015 - INSERM U1083, University of Angers MitoLab Team, University Hospital of Angers, Angers, France

**Keywords:** Genetic association study, Optic nerve diseases, Retinal diseases, Hereditary eye disease

## Abstract

Dominant optic atrophy (DOA) is genetically heterogeneous and most commonly caused by mutations in *OPA1*. To distinguish between the classical *OPA1*-related and the recently identified *SSBP1*-related DOAs, the retina and fovea of 27 patients carrying the *SSBP1* p.Arg38Gln variant were scrutinized using 20° × 20° macular cube and 30° and 55° field fundus autofluorescence photographs. Age of onset, visual acuity, retinal nerve fiber layer and macular thicknesses were recorded. Three *SSBP1-*patients were asymptomatic, 10 had isolated DOA, and 12 had a combined DOA plus foveopathy. The foveopathy, with a tiny defect of the ellipsoid and interdigitation lines, was similar in all patients, independent of age. There were no significant statistical differences in terms of visual acuity and SD-OCT measurements between patients with isolated DOA (mean visual acuity in decimals: 0.54 ± 0.41) and those with combined foveopathy (0.50 ± 0.23). Two patients over 50 years of age developed a progressive rod-cone dystrophy, leading to severe visual impairment. *SSBP1*-related DOA shares similarities with *OPA1*-related DOA with an incomplete penetrance and an early childhood visual impairment. Nevertheless, the presence of a congenital foveopathy with no impact on visual acuity is a major criterion to distinguish *SSBP1* cases and orient the appropriate genetic analysis.

## Introduction

Inherited optic neuropathies (ION) share a mitochondrial dysfunction despite a high variability in clinical presentation and genetic diagnoses^1–4^. IONs are caused by variants in mitochondrial DNA (mtDNA) genes, such as Leber Hereditary Optic Neuropathy (LHON, OMIM: 535000) or in nuclear genes with dominant or recessive transmissions. Dominant optic atrophy (DOA, OMIM: 165500) is the most frequent example of the latter group of IONs. DOA is characterized by bilateral mild chronic visual loss, starting typically during childhood, and 15% of mutation carriers are asymptomatic in OPA1 cases. OPA1 is the most common causative gene^2,4–11^ and encodes a GTPase involved in mitochondrial membrane fusion, cristae structure and mitochondrial genome maintenance^5,12–15^. Severe visual loss is rare in *OPA1*-related DOA, and generally associated with syndromic forms due to an accumulation of mtDNA deletions^1,4,10,15^. To date, no retinal alterations have been associated with *OPA1*-related DOA.

Since the discovery of *OPA1*, variants in other nuclear genes (*OPA3*, *DNM1L, MFN2, AFG3L2, SPG7, ACO2, NDUFS2, TMEM126A, RTN4IP1*) encoding mitochondrial proteins have been reported to cause DOA. More recently, we and others described missense *SSBP1* variants as a novel cause of DOA^16–20^. *SSBP1* (MIM:600439) encodes the mitochondrial single-stranded DNA-binding protein (Mt-SSB) involved in mitochondrial DNA replication^5–9^ together with other proteins of the mtDNA replisome, including twinkle mtDNA helicase (*TWNK*, OMIM: 606075), DNA polymerase G (*POLG*, OMIM:174763) and transcription factor A (*TFAM*, OMIM:600438)^21^. *SSBP1* individuals present a severe syndromic DOA with renal and neurological symptoms^16,17,19,20,22^. Furthermore, some patients were reported to have retinal alterations. Although the *SSBP1*-related DOA has been well documented, the frequency of asymptomatic carriers and of retinal alterations remain poorly estimated^16,17,19,20,22^.

Therefore, we clinically evaluated all individuals of the largest *SSBP1* family reported to date^16^. We re-examined the 21 initial patients and included 6 novel relatives carrying the *SSBP1* c.113G > A (p. Arg38Gln) variant in order to evaluate the frequency of asymptomatic carriers, as well as the cases of isolated DOA, of DOA plus foveopathy, and of DOA plus rod-cone dystrophy. Of importance, all patients underwent complete and careful analysis of the fovea by spectral domain ocular coherence tomography (SD-OCT), with multiple close iterative foveolar scans to avoid overlooking the foveopathy. Lastly, we statistically analyzed the impact of the foveopathy on the visual acuity and on the severity of the disorder.

## Results

### Clinical heterogeneity

**Figure 1:**
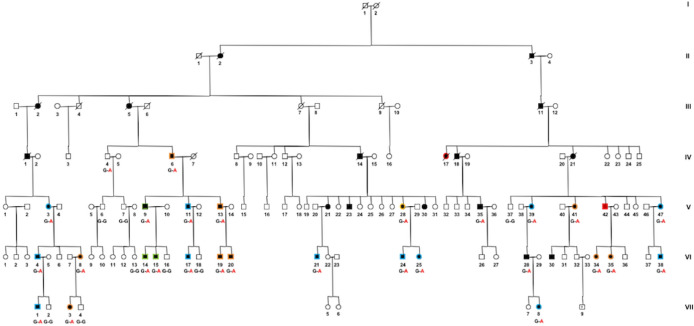
Pedigree of the large dominant optic atrophy family with the *SSBP1* c.113G > A variant. Isolated DOA = black symbols surrounded by an orange border. DOA plus foveopathy = black symbols surrounded by a blue border. DOA plus rod cone dystrophy = black symbols surrounded by a red border. Asymptomatic carriers = black symbols surrounded by a green border. Individual V:41 had a type 2 diabetes diagnosed at the age of 62, all other affected patients had no systemic disorder. This pedigree was drawn by B.B. using Cyrillic software version 2.1.3 (https://www.apbenson.com/software).

Among the 48 relatives from a single *SSBP1* family, 27 individuals carried the c.113G > A (p.Arg38Gln) pathogenic variant, and were thoroughly reassessed. Their clinical data are summarized in the supplementary Table S1. The pedigree of this family (Fig. 1) was previously partially described^16^, and the same mutation was also reported in 6 individuals from 2 English families (Table 1 for summary of the clinical data from the published *SSBP1* patients)^20^. Different ocular phenotypes ranged from asymptomatic carriers without optic and retinal lesions, to DOA associated with late-onset rod-cone dystrophy (RCD). None of the patients had extraocular symptoms, consistent with the observations on three other families carrying the p.Arg38Gln variant^16,20^.

**Table 1:** Summary of the clinical data from the published *SSBP1* patients.

	Asymptomatic carriers	Isolated OA	+ Foveopathy	+ Rod-cone dystrophy	Mutations/inheritance	Systematic features
This study	3	10	12	2	p.Arg38Gln, AD (27 patients)	No systematic features
Piro-Mégy et al.^16^	ND	0	2	0	p.Arg38Gln, AD (2 patients, family B)	No systematic features
	ND	4	0	0	p.Arg107Gln, AD (4 patients)	No systematic features
Jurkute et al.^20^	ND	5	3	3	p.Arg107Gln, AD (11 patients)	Renal insufficiency, hypothyroidism, diabetes
	ND	2	3	1	p.Arg38Gln, AD (6 patients, two families)	No systematic features
	ND	1	ND	ND	p.Ser141Asn, AD (1 patient)	No systematic features
Del Dotto et al.^19^	ND	0	1	1	p.Arg107Gln, AD de nova (2 patients)	Sensorineural deafness, renal insufficiency
	ND	0	0	1	p.Gly40Val, AD de nova (1 patient)	Sensorineural deafness, renal insufficiency
	ND	1	0	0	p.Glu111Gln, AD (1 patient)	No systematic features
	ND	0	3	0	p.Asn62Asp, AD (3 patients)	Mild kidney anomalies
	ND	0	0	1	p.Ile132Val biallelic, AR (1 patient)	Deafness, cardiomyopathy, nephropathy, ataxia, growth retardation
Gustafson et al.^17^	ND	0	0	1 ptosis ophthalmoplegia	p.Glu27Lys, AD de nova (1 patient)	Mitochondrial deletion syndrome: Leigh syndrome including deafness, bone narrow failure, nephropathy, ataxia, growth failure, endocrine deficiencies, metabolic strokes

*AD* autosomal dominant, *AR* autosomal recessive, *ND* not determined.

Among the 27 patients, 3 individuals aged 11, 15, and 51 years were strictly asymptomatic. Their visual acuity was 20/20 or better, with a normal optic nerve as determined by fundus examination and a normal retinal nerve fiber layer (RNFL) thickness by SD-OCT. The 24 symptomatic patients, 12 women and 12 men, had bilateral DOA, which was either isolated or combined with a retinal disorder. Visual loss occurred in early childhood, during the first decade for 16 patients or in the second decade for 4 (mean age of onset: 12.27 ± 10.31), whereas 4 other patients experienced an adult-onset from 29 to 37 years. Initial mean visual acuity was 20/50 (in decimals 0.434 ± 0.336). Visual acuity was above 20/40 in at least one eye in 13 patients. At the last ophthalmological assessment (mean follow-up: 16.84 years), six patients were legally blind (< 20/200), among which 2 since childhood, whereas visual acuity was above 20/40 in at least one eye in 9 patients.

### DOA phenotype

DOA was characterized by an optic disc pallor limited to the temporal neuroretinal rim associated with central, caeco-central or paracentral scotomas on kinetic perimetry and preserved peripheral isopters in all cases, except for the two individuals who displayed a RCD. Beyond the ocular involvement, there was no evidence of extraocular symptoms, such as progressive external ophthalmoplegia, hearing loss, renal or neurological disorders. Only one affected woman (V:41) developed a late onset type 2 diabetes at the age of 65 years.

Ten patients had an isolated DOA (Table S1 in supplemental digital content). Their visual loss occurred during the first two decades in all but two cases (mean 13 years ± 10.92). Visual acuity at baseline varied from 20/600 to 20/16, (mean in decimals: 0.54 ± 0.41) for a mean age of 20.9 years (± 13.70). One patient was legally blind (VI:35) since childhood. For the remaining nine patients: four patients had a visual acuity lower than 20/40 in both eyes, two a visual acuity of 20/40 in both eyes, and three a visual acuity of 20/20 or better in both eyes (VI:8, VI:19, VII:3). Lanthony D-15 test performed in eight patients was preserved in four cases and revealed a protan or deutan axes in three patients in at least one eye and a tritan axis in the last patient. The mean age at first SD-OCT analysis was 38.5 years ± 24.07 (10 to 83 years). Mean temporal RNFL thickness varied from 10 to 54 µm (mean 33 µm ± 12 µm). Foveal thickness varied from 133 to 217 µm (mean = 181 µm ± 34 µm), central (1 mm) thickness from 182 to 254 µm (mean = 227 µm ± 31 µm) and nasal 3 mm thickness from 237 to 320 µm (mean = 272 µm ± 24 µm). Evolution of visual acuity over time was documented in 7 patients (mean follow-up of 25.12 years) from initial 0.43 ± 0.33 to final 0.30 ± 0.34 mean visual acuity in decimals. At last examination, 2 additional patients were legally blind. Analysis of multimodal imaging progression data could not be achieved due to the restricted number of patients with initial and final SD-OCT follow-up. None of these patients developed a foveopathy.

### DOA plus foveopathy phenotype

**Figure 2:**
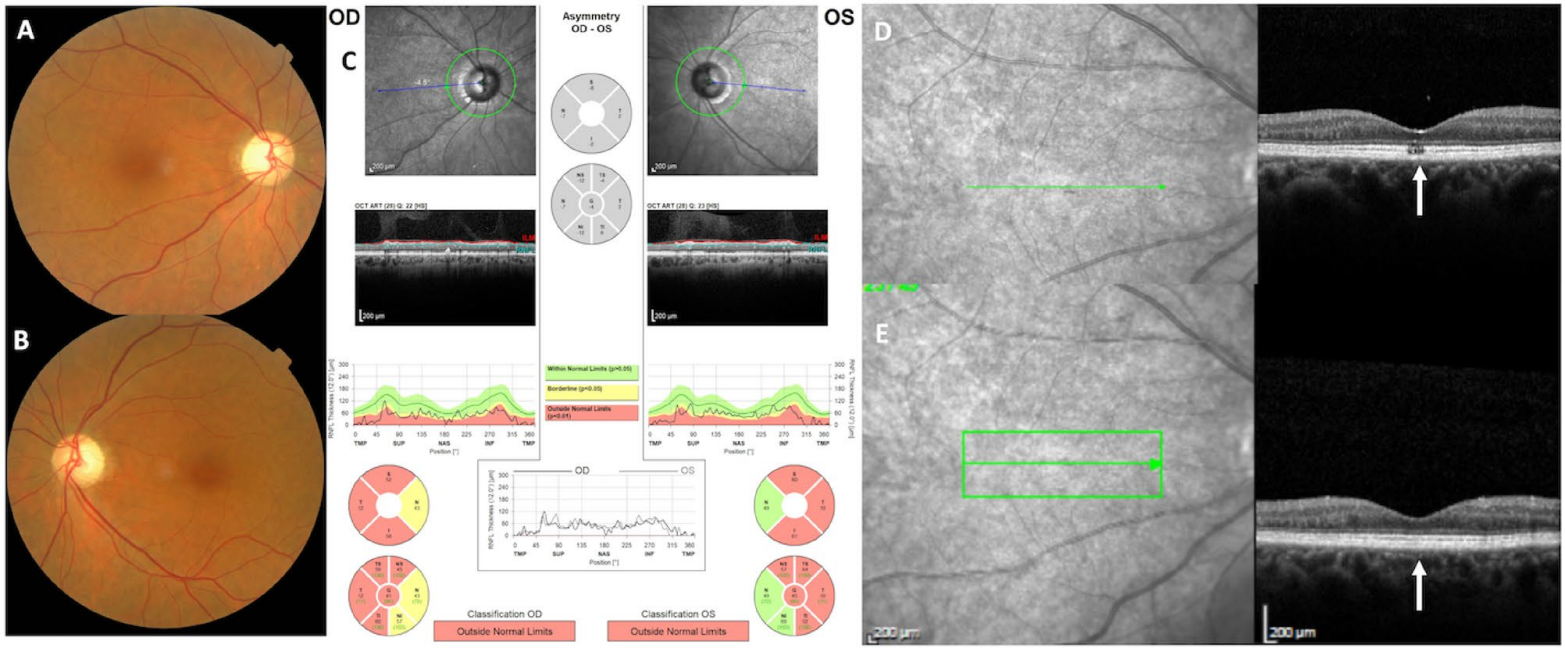
*SSBP1*-related DOA mimics *OPA1*-related DOA. Imaging analysis showing a temporal pallor (**A**, **B**) and significant bilateral thinning of the temporal retinal nerve fiber layer in both eyes (**C**). (**D**, **E**) Foveal SD-OCT scans in the right eye. In the absence of the specific foveopathy visible only by one SD-OCT scan (**D**), the optic atrophy is indistinguishable from the *OPA1*-related form. Photographs were taken by IM.

**Figure 3:**
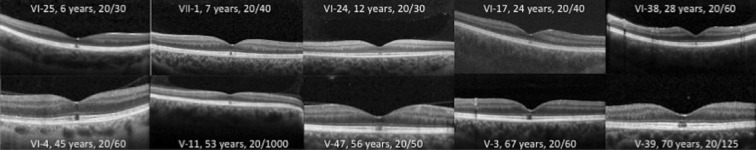
Unique foveopathy associated with *SSBP1*-related DOA. The *SSBP1*-related foveopathy is characterized by a tiny disruption of ellipsoid and interdigitation lines. Regardless of the age of the patient, the foveopathy always had the same appearance in ten different affected patients ranging in age from 6 to 70 years. Photographs were taken by IM.

**Figure 4:**
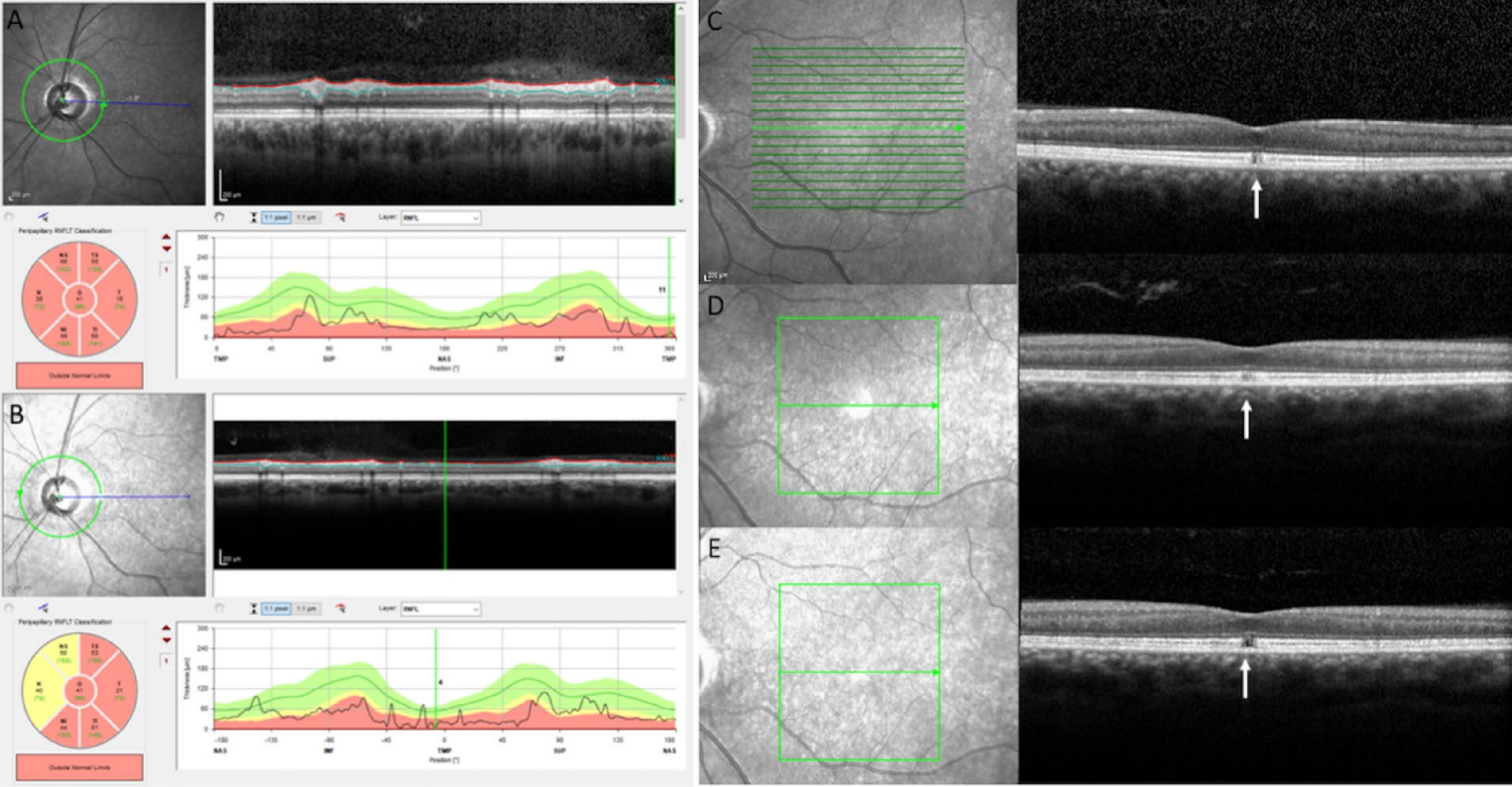
Stationary foveopathy in the individual V-11 with a 7 year SD-OCT follow-up. Visual acuity was severely decreased in both eyes, 20/400 at the age of 46 years and 20/800 at the age of 53 years. (**A**, **B**) RNFL thickness at initial (46 years) and final examinations (53 years) were severely reduced. (**C**–**E**) Infrared reflectance photographs and transfoveal scans of the patient at the age of 46 years (**C**), 50 (**D**) and 53 years (**E**) of the left eye. The foveal defect of the EZ and IZ lines was very small without any enlargement over time (white arrows). Photographs were taken by IM.

In addition to DOA, 12 *SSBP1* patients also had a foveopathy discovered by OCT examination. This foveopathy appeared as a tiny bilateral defect of the EZ and/or IZ, strictly restricted to the fovea (Figs. 2, 3, 4). On color pictures, a very small oblong pigment mottling was occasionally noticed. On fundus autofluorescence frames (FAF), the fovea and the macula were unremarkable without hypo nor hyper autofluorescence lesion.

Visual loss occurred during the first decade for 8 patients, in the second decade for 2, and later at 24 and 37 years for the remainder (mean age at onset 11.66 years ± 10.21). Visual acuity at baseline ranged from 20/400 to 20/25 with a mean value of 0.50 ± 0.23 in decimals for a mean age of 23.08 ± 21.12. One patient was legally blind (V:11). For the remaining 11 patients, 3 had a visual acuity less than 20/40 in both eyes, 1 had a visual acuity of 20/40 in one eye, and 7 had a visual acuity above 20/40 in both eyes. On Lanthony D-15 test performed on 11 patients, 4 showed a protan or deutan axis, four a tritan axis and 3 a normal color vision. The mean age at first SD-OCT was 33.08 years (± 21.34). Mean temporal RNFL thickness varied from 11 to 48 µm (mean 29 µm ± 11 µm). Foveal thickness varied from 149 to 204 µm (mean = 177 µm ± 15 µm), central (1 mm) thickness from 195 to 245 µm (mean = 218 µm ± 16 µm), and nasal 3 mm thickness from 247 to 314 µm (mean = 274 µm ± 20 µm). Evolution of visual acuity over time was documented in all patients (mean follow-up of 12 years, 2 to 41 years) from 0.5 (± 0.23) to 0.43 (± 0.22) in decimals, without reaching legal blindness. The associated foveopathy was never described in any other genetic optic neuropathy and had an invariable OCT pattern in all patients, regardless of age (Figs. 2, 3, 4). The lack of progression of the foveopathy is documented in patient V:11 with a HRA SD-OCT follow-up of seven years (Fig. 4).

### Isolated DOA versus DOA plus foveopathy

Isolated DOA and combined DOA plus foveopathy patients were comparable on demographic characteristics. For all the ophthalmological results, including visual acuity, RNFL, foveal, central macular measurements, no significant difference was observed between the two groups. SD-OCT parameters between the two groups are summarized in Table 2.

**Table 2:** Summary of SD-OCT parameters between the two groups: isolated dominant optic atrophy versus dominant optic atrophy plus foveopathy.

SD-OCT parameters	No foveopathy	Plus foveopathy	*P* value
RNFL temporal	33 µm ± 12 µm (10–54)	29 ± 11 µm (11–48)	> 0.05
Foveal thickness	181 ± 34 µm (133–217)	177 ± 15 µm (149–204)	> 0.05
Central (1 mm) thickness	227 ± 31 µm (182–254)	218 ± 16 µm (195–245)	> 0.05
Nasal 3 mm thickness	272 ± 24 µm (237–320)	274 ± 20 µm (247–314)	> 0.05

Female and male subjects were similar in terms of age of onset, visual acuity and RNFL thickness. But, females had a significant lower foveal and central macular thicknesses than males 163.91 ± 20.91 versus 193.45 ± 18.21, (*p* < 0.01) for foveal measurements and 210.61 ± 25.69 versus 230.59 ± 17.54, (*p* = 0.01) for central macular thickness.

Furthermore, affected patients did not have a smaller optic disk or an optic nerve hypoplasia whatever their ophthalmological presentation: isolated DOA or DOA + foveopathy, in comparison with 12 controls (24 eyes) as no significant difference in vertical and horizontal sizes were found.

### DOA plus RCD phenotype

**Figure 5:**
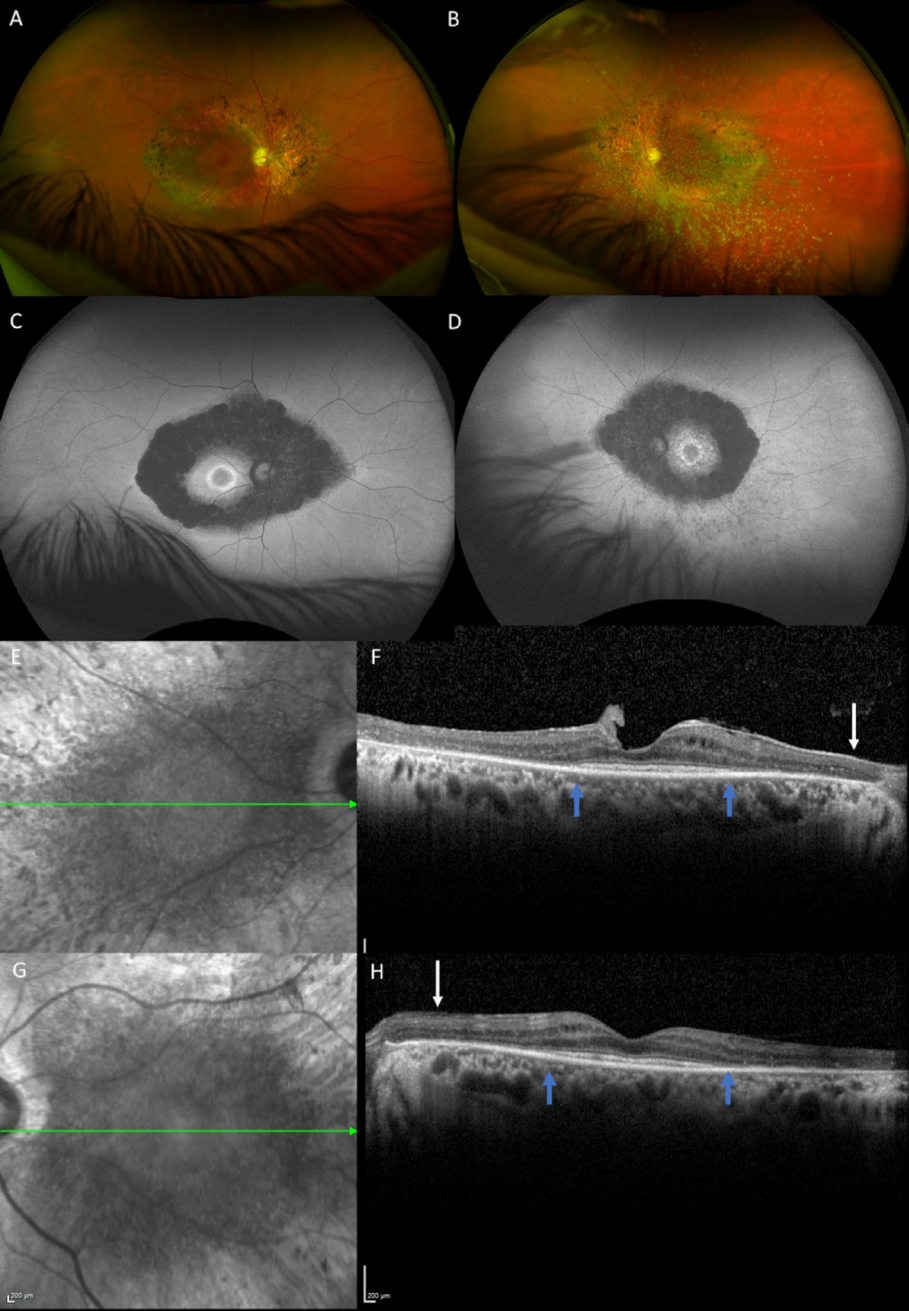
DOA plus rod-cone dystrophy in the individual V-42. This 69 year-old-patient was legally blind with a visual acuity of 20/400 in both eyes. Visual loss occurred at the age of 6 years. (**A**, **B**) Color fundus photographs. Note the temporal pallor and criteria of combined rod cone dystrophy with attenuated retinal vessels and typical pigmentary changes along the vascular arcades. (**C**, **D**) FAF showing patches of hypoautofluorescence more prominent and confluent around the vascular arcades, and an autofluorescence perifoveal ring. (**E**–**H**) Infrared reflectance photographs and macular SD-OCT. There is a severe thinning of the papillomacular bundle (white arrows) compatible with the optic atrophy. The outer nuclear layer EZ and IZ lines are not visible outside the macula (blue arrows) in both eyes due to the rod-cone dystrophy. Photographs were taken by IM.

Among the 6 individuals older than 60 years, 2 patients (IV:17 and V:42) examined at the age of 70 and 69 years, respectively, presented with a RCD. Their full-field electroretinogram (ERG) recordings, fundus autofluorescent (FAF) imaging, and macular SD-OCT analyses were in line with a rod-cone dystrophy (Fig. 5). Furthermore, despite performing multiple and iterative macular scans, these two patients showed no evidence of a foveopathy.

**Figure 6:**
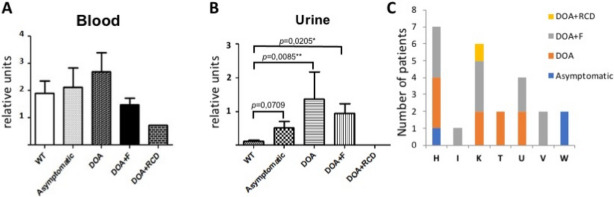
mtDNA quantification in blood (**A**) and urine (**B**) from controls (WT) and *SSBP1* individuals with the different ocular phenotypes. (**C**) Distribution of the *SSBP1* individuals with the different ocular phenotypes, according to their haplogroup.

In order to monitor if the status of the mtDNA could influence the different clinical phenotypes related to the *SSBP1* variant, we assessed the mtDNA copy number in blood and urine of control and *SSBP1* individuals, and determined their haplogroup. In blood samples, although no significant difference in mtDNA copy number was observed in relation to the clinical phenotype, a tendency towards increased amounts was observed in asymptomatic and isolated DOA patients, whereas a tendency to decreased mtDNA amounts was observed in patients more severely affected with DOA + foveopathy and DOA + RCD (Fig. 6A). In urine samples, all clinical categories associated to *SSBP1* individuals displayed a significantly increased mtDNA copy number compared to controls. Maximal levels were observed in patients with isolated DOA (Fig. 6B), thus suggesting that increased mtDNA copy number can modulate the severity of the clinical presentation associated with the *SSBP1* variant. Conversely, no specific haplogroup could be associated with the different *SSBP1* clinical presentations (Fig. 6C).

## Discussion

Although the DOA caused by *SSBP1* variants is highly similar to that caused by *OPA1* variants, we illustrate here that *SSBP1*-related DOA can be specifically distinguished by its association with a stationary foveopathy, which to date has never reported in any other optic atrophy or mitochondrial disease.

*SSBP1*- and *OPA1*-related dominant optic atrophies share common characteristics, including the early onset before the age of 10 years, bilateral and symmetrical mild to severe visual loss, and large intra- and inter-familial variability, including asymptomatic carriers. In this particular *SSBP1* family, visual acuity was highly variable ranging from 20/4000 to 20/16 (mean of 20/50), in asymptomatic (11.4% of individuals, 3/27) to legally blind (21.8% of individuals 6/27) individuals, respectively. In previously reported *OPA1* cohorts, asymptomatic carriers accounted for 4% to 18% of all cases, while disease onset occurred in 50% of cases before 20 years of age^1,2,6–11^. During the follow-up of *SSBP1* individuals, the visual acuity remained stable in 14 out of the 18, as is frequently described in most *OPA1* individuals. Similarly, the optic nerve pallor was restricted to the temporal quadrant, with temporal RNFL thickness ranging from 4 to 53 µm (mean 36.57 µm)^23^.

The similarities between *OPA1-* and *SSBP1*-associated DOA can be related to the involvement of both proteins in mtDNA replication and maintenance. Among the 8 OPA1 isoforms, 4 isoforms, including the alternative spliced exon 4b, are directly involved in mtDNA replication and distribution. The others are mandatory for the fusion and apoptotic activities, which are required for the kiss-and-run process involved in mitochondrial quality control^2,3,8,13–15^. *SSBP1* encodes a 148 amino-acid long single-strand binding mitochondrial protein with a key role in mtDNA replication, maintenance and repair. It forms tetramers enhancing the primer recognition and the binding of the single-stranded mtDNA at the replication fork, to promote efficient initiation and elongation of the replication forks by the POLG polymerase^13,16,19,20,24^. Importantly, the p.Arg38Gln variant decreases the abundancy of SSBP1 monomers and their capacity to form tetramers^16^, with deleterious consequences on mitochondrial genome replication. This leads to a 50% reduction in mtDNA levels in patient fibroblasts. Interestingly, SSBP1 is highly expressed during embryogenesis to promote RGC dendritogenesis and synaptogenesis, and retinal development^18^. During this developmental period, mitochondria accumulate at the neuronal growth cones to promote axon elongation and dendrite growth^25^. Thus, *SSBP1* foveopathy could thus be induced by a focal misrouting or a misalignment of the cone outer segments in the fovea, due to insufficient mitochondrial motility or energy production, during the initial stage of macular differentiation^26^.

Thus, although the alteration of the optic nerve and its consequences on visual performance are comparable between *SSBP1* and *OPA1* individuals, we disclose here that 44.4% of *SSBP1* individuals have a stationary foveopathy, and 7.4% a progressive RCD, combined with the optic nerve alterations (Fig. 5). The foveopathy is characterized by a tiny interruption of the EZ line and eventually of the IZ line (Figs. 2, 3, 4), which is only visible on one to two adjacent OCT scans. By contrast, it is not detectable on autofluorescence frames thus highlighting that the foveopathy can be underestimated. Based on the literature, the foveopathy was reported in 12 of 33 patients (36% of patients, Table 1) versus 12/27 (44% of cases) in our study. Per se, the foveopathy is most likely not responsible for the reduced visual acuity, as we did not find a significant difference in visual acuity between isolated DOA (0.54 or 20/40) and the DOA plus foveopathy (0.50 or 20/40) subgroups. During SD-OCT follow-up, the foveopathy did not enlarge with time and none of the patients without foveopathy at presentation developed such a lesion. The lack of impact on visual acuity and the unchanged pattern of the foveopathy with age suggest that it might represent a congenital defect, as previously proposed^19^. The presence of this unique foveopathy is thus a discriminating feature to diagnose a DOA as associated to *SSBP1*, and should also prompt the implementation of close iterative transfoveal SD-OCT scans to identify this defect (Fig. 3). It should be stressed that there is no report of such a foveal lesion in the most frequent *OPA1* forms, nor in all the optic neuropathies related to other genetic causes. In addition, the *SSBP1*-related foveopathy is the first stationary retinal lesion reported among all mitochondrial disorders. Along this line, in maternally inherited diabetes and deafness (MIDD syndrome), the characteristic macular lesions are more diffuse with a pattern-dystrophy appearance, which do progress after the third decade to macular atrophic lesions^4,27^. In mitochondrial pigmentary retinopathy related to the Kearns Sayre syndrome, a salt and pepper appearance is observed within the entire peripheral and central retina, and also progresses with time^4^. Similarly, toxic mitochondriopathies, such as the one related to long term antiretroviral therapy, generate retinal lesions mimicking a gyrate atrophy or a bull-eye maculopathy, which progress over time^4^.

A RCD is the second but less frequently associated retinal phenotype occurring in adulthood in 2 patients (7.4%) from our cohort, and was reported in 8 of 33 (24.24%) published *SSBP1* cases (Table 1). RCD is also encountered in *OPA1* “plus” and *ACO2* optic atrophies, and in mitochondrial diseases with mtDNA deletions, like Kearns Sayre syndrome^4^. The occurrence of a progressive RCD in *SSBP1* individuals could be explained by the age-related worsening of the mitochondrial genome depletion, as SSBP1 is required to stabilize single-stranded mtDNA and stimulate its replication by POLG^28^.

Lastly, none of the patients studied here displayed systemic features. Based on the *SSBP1* families published to date, the p.Arg38Gln variant only generated ocular symptoms, contrasting with the syndromic clinical presentations occurring with other *SSBP1* missense variants (Table 1). In this respect, the severity of the *SSBP1*-related diseases might be proportional to the residual mtDNA levels in patients. *SSBP1* variants could have different consequences on tetramer abundance and activity, which would lead to a gradient of mtDNA depletion, a parameter that must exceed 65% in affected organs to be considered as a mtDNA depletion syndrome^29^.

We can further hypothesize that secondary genetic and non-genetic determinants could account for the different clinical conditions described here. With regards to *SSBP1* involvement in mtDNA replication and maintenance, one can suppose that the quantity and integrity of the mitochondrial genome could contribute to modulation of the disease severity^30–32^. Results from mtDNA analyses among the different categories of *SSBP1* patients suggest that mtDNA amounts contribute to the clinical severity, as patients with isolated DOA displayed a tendency towards higher mtDNA copy number than those with DOA + foveopathy or RCD. Conversely, we could not associate a clinical presentation to a specific haplogroup, suggesting that the quantity but not the diversity of the mitochondrial genome might contribute to the severity of the clinical phenotype. This will need to be confirmed in other *SSBP1* patient cohorts, with further emphasis on syndromic individuals with extra-ophthalmological symptoms.

In conclusion, *SSBP1*-related DOA shares strong similarities with *OPA1*-related DOA, but is a unique disorder due to its frequent association with a congenital stationary foveopathy that is not per se a factor of vision loss. Few *SSBP1* patients are likely to develop a RCD in adulthood. Importantly, with 11% of asymptomatic carriers, genetic analysis is required in *SSBP1* family surveys to guarantee correct genetic counselling. Furthermore, it should then be recommended to systematically perform high resolution OCT scans centered on the fovea and large-field autofluorescence frames in all patients with DOA at their first visit and during follow-up to separate *SSBP1* cases from *OPA1* cases. Even if rare, the occurrence of a RCD during follow-up has to be explained to the patients in terms of additional central or peripheral visual loss and represents a further therapeutic challenge.

## Methods

### Patients

This retrospective cross-sectional study was approved by the Institutional Review Board of Montpellier University Hospital and the procedures used were in accordance with the tenets of the Declaration of Helsinki. Informed consent was obtained and signed by all patients after explanation of the nature and possible consequences of the study. Informed consent has been obtained to publish the information/image(s) in an online open-access publication.

### Clinical investigation

We re-examined all the related patients from our single French family carrying the pathogenic variant c.113G > A (p. Arg38Gln) in *SSBP1* gene. Age of onset, initial symptoms, best-corrected visual acuity with Snellen charts, 15-Hue desaturated color vision test, Goldmann visual fields, fundus photography (Nidek non-mydriatic automated fundus camera, AFC 330, Nidek Inc, Japan), infrared and autofluorescence imaging (Combined Heidelberg Retina Angiograph), spectral domain optical coherence tomography with retinal nerve fiber and macular analyses (Combined Heidelberg Retina Angiograph, Spectralis OCT device, Heidelberg Engineering, Dossenheim, Germany) were reviewed. The scan program used a 20° × 20° macular cube centered on the fovea included 97 high-resolution scans with 5 frames per scan and high-resolution horizontal scans with at least 50 frames per scan (ART mean). The foveal thicknesses and the automated ETDRS grid thicknesses (center, 1 mm, nasal 3 mm, superior 3 mm, temporal 3 mm, inferior 3 mm) were reviewed. The Heidelberg RNFL program was selected (3 acquisitions, circle 12°, ART 100). These tests allow us to correctly classify patients with the *SSBP1* pathogenic variant into four groups: (1) asymptomatic patients with no abnormalities, (2) patients with isolated neuropathy, (3) patients with neuropathy and foveopathy and iii. patients with RCD.

### Statistical analysis

The study population was described with means and standard deviations (SD) for quantitative variables and with frequencies for qualitative variables. The continuous variables distributions were tested using the Shapiro–Wilk test. Quantitative variables were compared using the Student’s t-test when the distribution was Gaussian and with the Mann–Whitney test, otherwise. For qualitative variables, groups were compared using the Χ^2^ test or Fisher’s exact test. The statistical significance was set at 0.05 and analyses were performed using Statistical Analysis Systems Enterprise Guide version 4.3 (SAS Institute, Cary, NC, USA). We compared patients with isolated DOA and patients with DOA plus foveopathy. Due to the low number of patients with DOA plus RCD, no statistical analysis could be performed for this subgroup.

## Supplementary Information

Supplementary Table S1.
