# Supplementary material for: Characterization of SSBP1-related optic atrophy and foveopathy
Source: Sci Rep. 2021 Sep 21;11:18703. doi: 10.1038/s41598-021-98150-1 (PMC8455542; doi:10.1038/s41598-021-98150-1)
Supplement: Supplementary file 1 — Supplementary Table S1. [file 41598_2021_98150_MOESM1_ESM.pdf]

Table 1: Clinical and imaging data of the 27 included patients carrying the *SSBP1* c.113G>A variant: from asymptomatic carriers to rod cone dystrophy.

Patient, Gender	Age of onset	VA RE/LE Age	Pallor	Initial RNFL TSNI RE/LE Age	Final RNFL TSNI RE/LE Age	Initial ETDRS Thickness RE-LE Foveal Central 1mm Nasal 3mm Sup 3mm Temp 3mm Inf 3mm Age	Final ETDRS Thickness RE-LE Foveal Central 1mm Nasal 3mm Sup 3mm Temp 3mm Inf 3mm Age	Phenotypes
IV:6, M	12	20/50-20/50<br>at 12 years<br><br>20/400-20/400<br>at 83 years.	T	No SD-OCT<br>at baseline	T 33/39<br>S 60/110<br>N 52/88<br>I 76/53<br>At 83 years	No SD-OCT<br>at baseline	F208-209<br>C241-235<br>N266-254<br>S257-257<br>T274-256<br>I262-253<br>At 83 years	DOA<br>AMD drusen
V:13, M	6	20/40-20/40<br>at 30 years<br><br>20/60 -20/50<br>at 46 years	T	No SD-OCT<br>at baseline	T 32/NA<br>S 33/NA<br>N 48/NA<br>I 69/NA<br>At 46 years	No SD-OCT<br>at baseline	F203-215<br>C240-267<br>N260-263<br>S233-259<br>T266-273<br>I279-292<br>At 46 years	DOA
V:28, F	30	20/25-20/32<br>at 33 years<br><br>20/50-20/40<br>at 45 years	T	No SD-OCT<br>at baseline	T 49/53<br>S 106/90<br>N 58/68<br>I 100/119<br>At 45 years	No SD-OCT<br>at baseline	F141-135<br>C185-179<br>N257-251<br>S255-244<br>T249-246<br>I254-271<br>At 45 years	DOA
V:41, F	6	20/200-20/200<br>at 15 years<br><br>20/4000-20/1000<br>At 67 years	T	No SD-OCT<br>at baseline	T 17/4<br>S 57/51<br>N 41/36<br>I 52/47<br>At 67 years	No SD-OCT<br>at baseline	F137-128<br>C191-189<br>N246-228<br>S233-229<br>T224-221<br>I237-227<br>At 67 years	DOA

VI:8, F	36	20/20-20/20<br>at 36 years	T	T 34/37<br>S 77/84<br>N 51/50<br>I 91-83<br>At 36 years	No follow-up	F185-196<br>C239-247<br>N285-289<br>S286-291<br>T281-285<br>I280-283<br>At 36 years	No follow-up	DOA
VI:19, M	12	20/16-20/16<br>at 12 years	T	T 34/36<br>S 123/111<br>N 79/85<br>I 130/97<br>At 12 years	No follow-up	F217-216<br>C249-247<br>N280-279<br>SNR-NR<br>T280-277<br>UD-UD<br>At 12 years	No follow-up	DOA
VI:20, M	6	20/63-20/63<br>at 6 years<br><br>20/80-20/100<br>at 10 years	T	No SD-OCT<br>at baseline	T 25/24<br>S 96/90<br>N 52/47<br>I 81/79<br>At 10 years	No SD-OCT<br>at baseline	F217-212<br>C245-243<br>N287-286<br>S286-286<br>T280-276<br>I287-286<br>At 10 years	DOA
VI:34, F	6	20/125-20/125<br>at 45 years	T	No SD-OCT<br>at baseline	T 23/24<br>S 72/68<br>N 66/64<br>I 81/68<br>At 45 years	No SD-OCT<br>at baseline	F153-152<br>C187-187<br>N263-260<br>S259-256<br>T253-250<br>I255-253<br>At 45 years	DOA
VI:35, F	6	20/600-20/600<br>at 10 years<br><br>20/600-20/600<br>at 28 years	T	No SD-OCT<br>at baseline	T 33/33<br>S 44/28<br>N 27/22<br>I 39/46<br>At 28 years	No SD-OCT<br>at baseline	F155-146<br>UD<br>At 28 years	DOA

VII:3, F	10	20/20-20/20<br>at 10 years<br><br>20/20-20/20<br>at 13 years	T	T 58/49<br>S 121/121<br>N 76/72<br>I 127/130<br>At 10 years	T 53/40<br>S 114/122<br>N 55/61<br>I 130/132<br>At 13 years	F 200-200<br>Cube not done<br>At 10 years	F197-196<br>C255-253<br>N320-320<br>S328-327<br>T323-321<br>I324-323<br>At 13 years	DOA
V:3, F	8	20/50-20/50<br>at 65 years<br><br>20/50-20/50<br>at 68 years	T	No SD-OCT<br>at baseline	T 25/26<br>S 67/68<br>N 52/49<br>I 70/64<br>At 68 years	No SD-OCT<br>at baseline	F152-147<br>C214-209<br>N273-270<br>S271-267<br>T264-261<br>I267-263<br>At 68 years	DOA+F
V:11, M	6	20/400-20/400<br>at 46 years<br><br>20/800-20/800<br>at 50 years<br><br>20/800-20/800<br>at 53 years	T	T 10/16<br>S 59/59<br>N 40/40<br>I 46/48<br>At 46 years	T 10/21<br>S 59/59<br>N 40/40<br>I 46/48<br>At 53 years	F 200-201<br>C 231-235<br>N 256-261<br>S 249-250<br>T 242-244<br>I 248-251<br>At 46 years	F203-215<br>C230-238<br>N255-259<br>S246-251<br>T241-245<br>I247-251<br>At 53 years	DOA+F
V:39, F	37	20/160-20/160<br>at 56 years<br><br>20/160-20/160<br>at 72 years	T	No SD-OCT<br>at baseline	T 12/10<br>S 52/60<br>N 41/49<br>I 58/61<br>At 72 years	No SD-OCT<br>at baseline	F161-175<br>C192-198<br>N247-247<br>UD-UD<br>T235-231<br>UD-UD<br>At 72 years	DOA+F

V:47, F	16	20/32-20/32<br>At 16 years<br><br>20/32-20/32<br>At 60 years	T	No SD-OCT<br>at baseline	T 52/46<br>S 106/115<br>N 87/89<br>I 116/110<br>At 60 years	No SD-OCT<br>at baseline	F186-181<br>C235-230<br>N315-313<br>S305-308<br>T303-303<br>I309-308<br>At 60 years	DOA+F
VI:4, M	9	20/32-20/32 at 11 years<br><br>20/63- 20/40<br>at 48 years	T	T 27/26<br>S 71/70<br>N 43/43<br>I 91/76<br>At 45 years	T 26/27<br>S 72/72<br>N 42/43<br>I 90/79<br>At 48 years	F198-207<br>C240-248<br>N287-291<br>S288-292<br>T286-286<br>I283-286<br>At 45 years	F204-214<br>C246-251<br>N290-293<br>S290-294<br>T286-287<br>I284-285<br>At 48 years	DOA+F
VI:17, M	3	20/40-20/40<br>at 22 years<br><br>20/40-20/50 at 25 years	T	T 26/26<br>S 65/87<br>N 50/44<br>I 110/113<br>At 22 years	T 28/26<br>S 64/88<br>N 49/42<br>I 113/110<br>At 25 years	F176-177<br>C218-223<br>N285-285<br>S271-272<br>T267-269<br>I276-275<br>At 22 years	F173-184<br>C219-225<br>N287-286<br>S273-273<br>T268-272<br>I277-278<br>At 25 years	DOA+F
VI:21, M	24	20/25-20/25<br>At 24 years<br><br>20/32-20/32<br>At 30 years	T	No SD-OCT<br>at baseline	T 36/38<br>S 90/105<br>N 68/65<br>I 86/93<br>At 30 years	No SD-OCT<br>at baseline	F175-174<br>C208-205<br>N261-264<br>S258-264<br>T254-256<br>I260-258<br>At 30 years	DOA+F
VI:24, M	4	20/32-20/32 at 4 years<br><br>20/32-20/32 at 11 years	T	No SD-OCT<br>at baseline	T 30/27<br>S 113/110<br>N 62/73<br>I 107/108<br>At 11 years	No SD-OCT<br>at baseline	F164-16<br>C205-203<br>N257-255<br>S256-259<br>T255-255<br>I256-255<br>At 11 years	DOA+F

VI:25, F	5	20/25-20/25<br>At 5 years<br><br>20/25-20/25<br>At 14 years	T	No SD-OCT<br>at baseline	T 40/39<br>S 97/110<br>N 74/70<br>I 113/108<br>At 14 years	No SD-OCT<br>at baseline	F169-170<br>C202-199<br>N269-269<br>UD-UD<br>T262-259<br>UD-UD<br>At 14 years	DOA+F
VI:38, M	17	20/32-20/32<br>at 17 years<br><br>20/63-20/50<br>at 28 years	T	No SD-OCT<br>at baseline	T 24/26<br>S 86/82<br>N 62/47<br>I 77/86<br>At 28 years	No SD-OCT<br>at baseline	F183-179<br>C234-232<br>N293-294<br>S282-287<br>T288-293<br>I282-287<br>At 28 years	DOA+F
VII:1, M	5	20/50-20/50<br>at 6 years<br><br>20/50-20/50<br>at 8 years	T	T 40/44<br>S 107/104<br>N 76/74<br>I 102/125<br>At 6 years	ND at 8 years	ND at 6 years	F179-182<br>C216-208<br>N274-265<br>S269-266<br>T269-263<br>I277-269<br>At 8 years	DOA+F
VII:8, F	6	20/40-20/50<br>at 6 years<br><br>20/125-20/100<br>at 11 years	T	No SD-OCT<br>at baseline	RNFL unrealizable	No SD-OCT<br>at baseline	F 169-175<br>UD	DOA+F
IV:17, F	5	20/400-20/400<br>at 70 years	T	NA OCT-3	Dead	NA OCT-3	dead	DOA+RCD
V:42, M	6	VA < 20/40<br>at the age of 18.<br><br>20/400-20/400<br>at 69 years	T	No SD-OCT	T 15/UD<br>S 40/UD<br>N 5/UD<br>I 66/UD<br>At 69 years	No SD-OCT	F136-156<br>C223-197<br>N285-249<br>S263-231<br>T247-225<br>I256-229<br>At 69 years	DOA+RCD

V:9, M		20/20-20/20<br>At 51 years	No	T 69/71<br>S 131/140<br>N 54/54<br>I 122/115<br>At 51 years	No follow-up	F195-197<br>C246-246<br>N303-300<br>S301-302<br>T289-286<br>I296-295<br>At 51 years	No follow-up	Asymptomatic
VI:14, M		20/20-20/20<br>At 15 years	No	T 79/73<br>S 134/142<br>N 77/89<br>I 149/140<br>At 15 years	No follow-up	F254-232<br>No macular cube	No follow-up	Asymptomatic
VI:15, M		20/20-20/20<br>At 11 years	No	T 79/74<br>S 140/141<br>N 104/96<br>I 146/151<br>At 11 years	No follow-up	F237-233<br>C285-282<br>N355-355<br>S349-346<br>T341-341<br>I355-356<br>At 11 years	No follow-up	Asymptomatic

DOA: dominant optic atrophy. DOA+F: dominant optic atrophy and combined foveopathy. DOA+RCD: dominant optic atrophy and combined rod cone dystrophy. LE: Left eye. ND: not done. RE: right eye. RNFL: retinal nerve fiber layer. UD: unreliable data.
